# The prognostic value of cachexia index in patients with lung cancer treated with immune checkpoint inhibitors: a propensity score matching analysis

**DOI:** 10.3389/fnut.2026.1810531

**Published:** 2026-07-20

**Authors:** Huachi Li, Mengxing Tian, Wen Wang, Qian Han, Xin Jin, Xiantian Xu

**Affiliations:** 1 Department of Gastrointestinal Surgery, Hubei Cancer Hospital, Tongji Medical College, Huazhong University of Science and Technology, Wuhan, Hubei, China; 2 Department of Clinical Nutrition, Hubei Cancer Hospital, Tongji Medical College, Huazhong University of Science and Technology, Wuhan, Hubei, China; 3 Department of Nutrition, Henan Provincial People’s Hospital, Zhengzhou University People’s Hospital, Zhengzhou, Henan, China; 4 Cancer Center, Henan Provincial People’s Hospital, Zhengzhou University People’s Hospital, Zhengzhou, China

**Keywords:** cachexia index, cancer cachexia, immune checkpoint inhibitors, lung cancer, overall survival

## Abstract

**Background:**

Cancer cachexia index (CXI) is a potential tool for cancer cachexia diagnosis in patients with lung cancer. However, the predictive value of the CXI for clinical outcomes in patients with lung cancer receiving immune checkpoint inhibitors (ICIs) remains unclear. This study aimed to investigate the role of the CXI in predicting overall survival in patients with lung cancer undergoing ICIs.

**Methods:**

A single-center retrospective study was conducted, enrolling 119 patients with lung cancer who received ICIs. The clinical, laboratory, abdominal computed tomography (CT) images and follow-up data before immunotherapy were collected for all participants. The CXI was calculated by integrating the CT-derived skeletal muscle index (SMI) at the third lumbar vertebra level with serum albumin and the neutrophil-to-lymphocyte ratio. Kaplan-Meier survival analysis was used to compare overall survival (OS) differences between patients with a low CXI and those with a high CXI. Univariate and multivariable Cox proportional hazards regression models were applied to identify independent prognostic factors for OS. Propensity score matching (PSM) was performed to minimize selection bias, and a nomogram was constructed based on the CXI.

**Results:**

Kaplan-Meier survival curves showed that patients with a low CXI had significantly lower 3-year OS rate than those with a high CXI in both the total cohort (24.6% vs. 65.1%, *p* < 0.001) and the PSM-matched cohort (23.2% vs. 74.2%, *P* = 0.001). Multivariable Cox regression analysis identified a low CXI as negative prognostic factor for OS in patients with lung cancer receiving ICIs in the total cohort (hazard ratio [HR] = 3.11; 95% confidence interval [CI]: 1.52–6.37; *p* < 0.001) and the PSM cohort (HR = 5.55; 95% CI: 1.86–16.61; *p* < 0.001). Gender, age, disease stage, smoking status, nutritional risk, BMI, pathology, type of ICIs, surgery and CXI were used to develop the nomogram. Additionally, the CXI was found to correlate with the SMI with a statistically significant weak to moderate positive correlation (ρ = 0.328; *p* = 0.0003).

**Conclusion:**

In patients with lung cancer receiving ICIs, CXI is an independent prognostic factor for OS. It may serve as a valuable clinical tool for assessing cancer cachexia in lung cancer patients undergoing immunotherapy.

## 1 Introduction

Lung cancer was the most commonly diagnosed cancer globally, with about 2.48 million new cases estimated in 2022. It was also the leading cause of cancer-related mortality, accounting for 18.7% of all cancer deaths (1). Moreover, lung cancer survival remains poor, with 5-year survival rates of 28% for non-small cell lung cancer (NSCLC) and 7%–9% for small cell lung cancer (SCLC), especially in patients with late-stage disease. Due to advances in treatment including immunotherapy, notable improvements in clinical outcomes have been achieved with lung cancer (2). Finding from KEYNOTE-024 showed that immunotherapy alone could outperform chemotherapy, with a median overall survival of 30 months for pembrolizumab compared with 14.2 months for chemotherapy (3). Key findings from the CheckMate trial demonstrated that among patients with programmed cell death ligand 1 (PD-L1) expression ≥1%, nivolumab combined with ipilimumab significantly improved median overall survival. However, the effects of immunotherapy also show significant interindividual variability. Although PD-L1 expression and Tumor Mutational Burden (TMB) are predictors of response to immunotherapy in lung cancer, they are not binary switches for success. In addition, treatment regimens developed based on these biomarkers may not be suitable for the overall populations (4). Therefore, additional predictive factors need to be identified to improve prognostic assessment of patients with lung cancer.

Cancer cachexia is a multifactorial syndrome defined by an international consensus as an ongoing loss of skeletal muscle mass (with or without loss of fat mass). This syndrome cannot be fully reversed by conventional nutritional support and results in progressive functional impairment (5). A systematic review and meta-analysis containing 125 articles showed that the overall prevalence of cachexia in patients with cancer was 33.0%. Numerous studies have confirmed that cancer cachexia is a stronger predictor of overall survival. According to Fearon 2011 criteria, patients with cancer cachexia had shorter overall survival than patients with non-cachexia (Hazard Ratio [HR] = 1.58) (6). Retrospective studies showed that the prevalence of cachexia was 28% at cancer diagnosis in patients with NSCLC (7), and these patients had worse survival than those without cachexia (8, 9). However, the lack of standardized and quantifiable criteria limits the broad application of cachexia diagnosis in lung cancer. The cancer cachexia index (CXI), which is calculated using skeletal muscle index (SMI), albumin (ALB) and neutrophil to lymphocyte ratio (NLR) is a potential index to diagnose cancer cachexia. Previous studies have shown that CXI is a predictor of prognosis in various types of cancer (10). However, the prognostic value of CXI in patients with lung cancer treated with immune checkpoint inhibitors remains uncertain.

Therefore, a retrospective study was conducted to explore the relationship between CXI and survival in patients with lung cancer treated with programmed cell death protein 1 (PD-1) and PD-L1 inhibitors. In this study, pretreatment CXI was analyzed and its association between pretreatment CXI and overall survival was investigated. In addition, a nomogram based on CXI was created to predict overall survival (OS) in patients with NSCLC treated with PD-1 and PD-L1 inhibitors. The relationship between CXI and SMI was also explored. To our knowledge, this is the first study to explore the predictive role of CXI in patients with lung cancer treated with immune checkpoint inhibitors (ICIs).

## 2 Materials and methods

### 2.1 Study design and patient selection

This was a single-center retrospective study conducted at Henan Province Hospital. The patients with lung cancer who met the inclusion and exclusion criteria and were treated at this hospital between May 2018 and October 2020 were enrolled. Patients were included if they met all of the following inclusion criteria: (1) had histopathological confirmed lung cancer; (2) were treated with immunotherapy agents such as PD-1 and PD-L1 inhibitors; (3) were aged between 18 and 80 years at the time of recruitment; (4) had undergone abdominal computed tomography (CT) scan with results available within 2 weeks before treatment commencement; and (5) provided complete clinical datasets. Patients were excluded if they met any of the following criteria: (1) had previously undergone a full course of immunotherapy; (2) lacked comprehensive medical records; or (3) were diagnosed with multiple primary malignancies. This retrospective study was approved by the Hospital Ethics Committee of Henan Provincial People’s Hospital (approval number: 2022-220), and the requirement for informed consent was waived given its retrospective design.

### 2.2 Immunotherapy regimen

All enrolled patients diagnosed with TNM stage II-IV lung cancer received immunotherapy regimens centered on PD-1/PD-L1 inhibitors as the primary treatment. Administration of PD-1 and PD-L1 inhibitors was performed in strict accordance with established guidelines and protocols for lung cancer management. Additionally, eligible patients received concurrent chemotherapy or targeted therapeutic agents.

### 2.3 Data collection, and follow-up

The pretreatment clinical characteristics including gender, age, height, weight, TNM stage, type of histology, smoking history and nutritional status were collected by a trained researcher. The type of ICIs and history of surgery were also collected from the medical records. All patients were routinely followed up every 3 months after discharge. Follow-up methods adopted in this study comprised telephone consultations, face-to-face outpatient visits, and hospital admissions for treatment. The last follow-up time was conducted on September 30, 2023. OS was defined as the time from initiation of immunotherapy to death from any cause. Patients alive at the final follow-up were censored.

### 2.4 Muscle mass measurement and cachexia index

Muscle mass was assessed using abdominal CT scans acquired with a GE Healthcare scanner within 2 weeks prior to the initiation of immunotherapy. A CT image at the level of the third lumbar vertebra (L3) was selected to measure muscle mass. Muscle mass was measured using software developed by Wenzhou Medical University, which utilizes convolutional neural networks for image processing. Skeletal muscle was identified according to predefined Hounsfield unit (HU) thresholds, with a range of −29 to 150 HU delineating muscle tissue. The skeletal muscle index (SMI) was subsequently calculated as muscle area normalized to the square of patient height (cm^2^). Neutrophil count, total lymphocyte count, and serum ALB levels were obtained from routine hematological and biochemical tests performed prior to the initiation of immunotherapy. NLR was calculated as neutrophil count/total lymphocyte count. The CXI was calculated as SMI × ALB/NLR.

### 2.5 Statistical analysis

All analyses were performed using R software (version 4.5.5). The continuous variables with normal distribution were expressed as mean and standard deviation, whereas those with a non-normal distribution were expressed as median and interquartile range (IQR), Categorical variables were expressed as frequencies and percentages. The optimal cutoff values were determined by the time-dependent receiver operating characteristic (ROC) analysis. Propensity score matching (PSM) was performed to balance age and nutritional risk between groups, resulting in a well-adjusted cohort. The Kaplan-Meier curves were performed to show the survival times of different groups. And the log-rank test was used to analyze difference between the curves. Univariate Cox proportional hazards analyses were conducted to investigate the relationship between prognostic factors and OS. A multivariate Cox proportional hazards regression model which including all the relevant factors was used to estimate HRs and 95% Confidence Intervals (CIs). A nomogram was developed to visualize the prognostic impact of multivariate predictors for OS. *P*-values < 0.05 were defined as statistically significant.

## 3 Results

### 3.1 Patient characteristics

One hundred and nineteen patients with lung cancer treated with ICIs who met the inclusion and exclusion criteria were included in this study. The median age of the cohort was 64 years (IQR, 53–70), with a median survival time of 28 months (IQR, 25–29). In the total cohort, 99 patients were diagnosed with NSCLC and 20 with SCLC. Nutritional risk (≥3 points) was assessed using the NRS 2002 tool in 77 patients. At the initiation of immunotherapy, 91 patients had stage IV disease, while 28 had stage II-III disease.

According to the results of the time-dependent ROC Analysis, the optimal cutoff value of CXI in this cohort was 404.3. Patients were divided into low CXI and high CXI groups based on this cutoff value. (Supplementary Figure 1) A total of 40 patients were classified into the low-CXI group (33.6%; 25 males and 15 females), and 79 patients into the high-CXI group (66.4%; 54 males and 25 females). The median age was 67.5 years in the low-CXI group and 61.0 years in the high-CXI group, with no significant difference. The patients with low CXI had lower SMI, ALB and NLR compared with the patients with high CXI. The patients with high CXI had lower incidence of nutritional risk accessed by Nutritional Risk Screening 2002 (NRS2002) (*P* = 0.039). No significant difference was observed in gender, BMI and type of pathology between these two groups. Considering the differences in age and the incidence of nutritional risk between the two groups, we conducted 1:1 propensity score matching. As a result, 35 participants were included in each group, forming a PSM cohort, as detailed in Table 1.

**TABLE 1:** Baseline characteristics of the total cohort and propensity score matching cohort.

		Total cohort			PSM cohort		
Characteristic	Level	Low CXI (*n* = 40)	High CXI (*n* = 79)	*P*	Low CXI (*n* = 35)	High CXI (*n* = 35)	*P*
Age (median [IQR])		67.5 [58.0, 70.0]	61.0 [53.0, 69.5]	0.079^†^	65.0 [58.0, 70.0]	65.0 [58.0, 70.0]	0.991^†^
Gender *n*, (%)	Female	15 (37.5)	25 (31.6)	0.665^#^	14 (40.0)	9 (25.7)	0.309^#^
	Male	25 (62.5)	54 (68.4)		21 (60.0)	26 (74.3)	
Smoking *n*, (%)	No	18 (45.0)	41 (51.9)	0.479^#^	17 (48.6)	19 (54.3)	0.811^#^
	Yes	22 (55.0)	38 (48.1)		18 (51.4)	16 (45.7)	
Stage *n*, (%)	II-III	10 (25.0)	18 (22.8)	0.968^#^	8 (22.9)	9 (25.7)	0.999^#^
	IV	30 (75.0)	61 (77.2)		27 (77.1)	26 (74.3)	
T stage *n*, (%)	1	2 (5.0)	12 (15.2)	0.157^#^	2 (5.7)	6 (17.1)	0.231
	2	9 (22.5)	15 (19.0)		9 (25.7)	9 (25.7)	
	3	6 (15.0)	20 (25.3)		4 (11.4)	7 (20.0)	
	4	22 (55.0)	28 (35.4)		20 (57.1)	13 (37.1)	
	X	1 (2.5)	4 (5.1)		0 (0.0)	0 (0.0)	
N stage *n*, (%)	0	0 (0.0)	1 (1.3)	0.084^#^	0 (0.0)	0 (0.0)	0.145^#^
	1	1 (2.5)	11 (13.9)		0 (0.0)	3 (8.6)	
	2	21 (52.5)	43 (54.4)		18 (51.4)	20 (57.1)	
	3	18 (45.0)	21 (26.6)		17 (48.6)	11 (31.4)	
	X	0 (0.0)	3 (3.8)		0 (0.0)	1 (2.9)	
M stage *n*, (%)	0	10 (25.0)	20 (25.3)	0.999^#^	8 (22.9)	9 (25.7)	0.999^#^
	1	30 (75.0)	59 (74.7)		27 (77.1)	26 (74.3)	
Type of pathology *n*, (%)	NSCLC	36 (90.0)	63 (79.7)	0.249^#^	31 (88.6)	28 (80.0)	0.513*
	SCLC	4 (10.0)	16 (20.3)		4 (11.4)	7 (20.0)	
BMI [mean (SD)]		22.95 (3.78)	23.61 (2.80)	0.281^‡^	23.52 (3.54)	24.04 (3.07)	0.511^‡^
Surgery *n*, (%)	No	36 (90.0)	70 (88.6)	0.999*	31 (88.6)	31 (88.6)	0.999*
	Yes	4 (10.0)	9 (11.4)		4 (11.4)	4 (11.4)	
Type of ICIs *n*, (%)	PD-1	38 (95.0)	75 (94.9)	0.999*	33 (94.3)	32 (91.4)	0.999*
	PD-L1	2 (5.0)	4 (5.1)		2 (5.7)	3 (8.6)	
ALB (median [IQR])		36.6 [33.5, 41.9]	40.8 [38.1, 43.6]	**0.001** ^†^	38.20 [33.10, 42.10]	40.80 [37.80, 43.40]	**0.048** ^†^
NLR (median [IQR])		5.69 [4.97, 7.75]	2.32 [1.67, 3.28]	<**0.001**^†^	5.49 [4.97, 7.49]	2.22 [1.55, 3.07]	<**0.001**^†^
CXI (median [IQR])		287.3 [200.5, 323.3]	786.4 [558.4, 1140.3]	**<0.001** ^†^	294.7 [208.5, 325.2]	822.9 [611.1, 1192.7]	<**0.001**^†^
SMI [mean (SD)]		43.17 (7.37)	48.24 (8.11)	**0.001** ^‡^	43.63 (7.44)	48.60 (7.77)	**0.008** ^‡^
NRS2002 *n*, (%)	Positive	17 (42.5)	60 (24.1)	**0.039** ^#^	12 (34.3%)	12 (34.3%)	0.999^#^
	Negative	23 (57.5)	19 (75.9)		23 (65.7%)	23 (65.7%)	

† Mann-Whitney U test; ^‡^T test; ^#^Chi square test; *Fisher’s extract test. PSM, propensity score matching; BMI, body mass index; ALB, albumin; NLR, neutrophil to lymphocyte ratio; SMI, skeletal muscle index CXI, cachexia index; SCLC, small cell lung cancer; NSCLC, non-small-cell lung cancer; ICIs, immune checkpoint inhibitors; PD-L1, programmed cell death ligand 1; PD-1, programmed cell death protein 1; NRS2002, Nutritional Risk Screening 2002. The statistically significant values were formatted in bold.

### 3.2 The effect of CXI on OS

**FIGURE 1:**
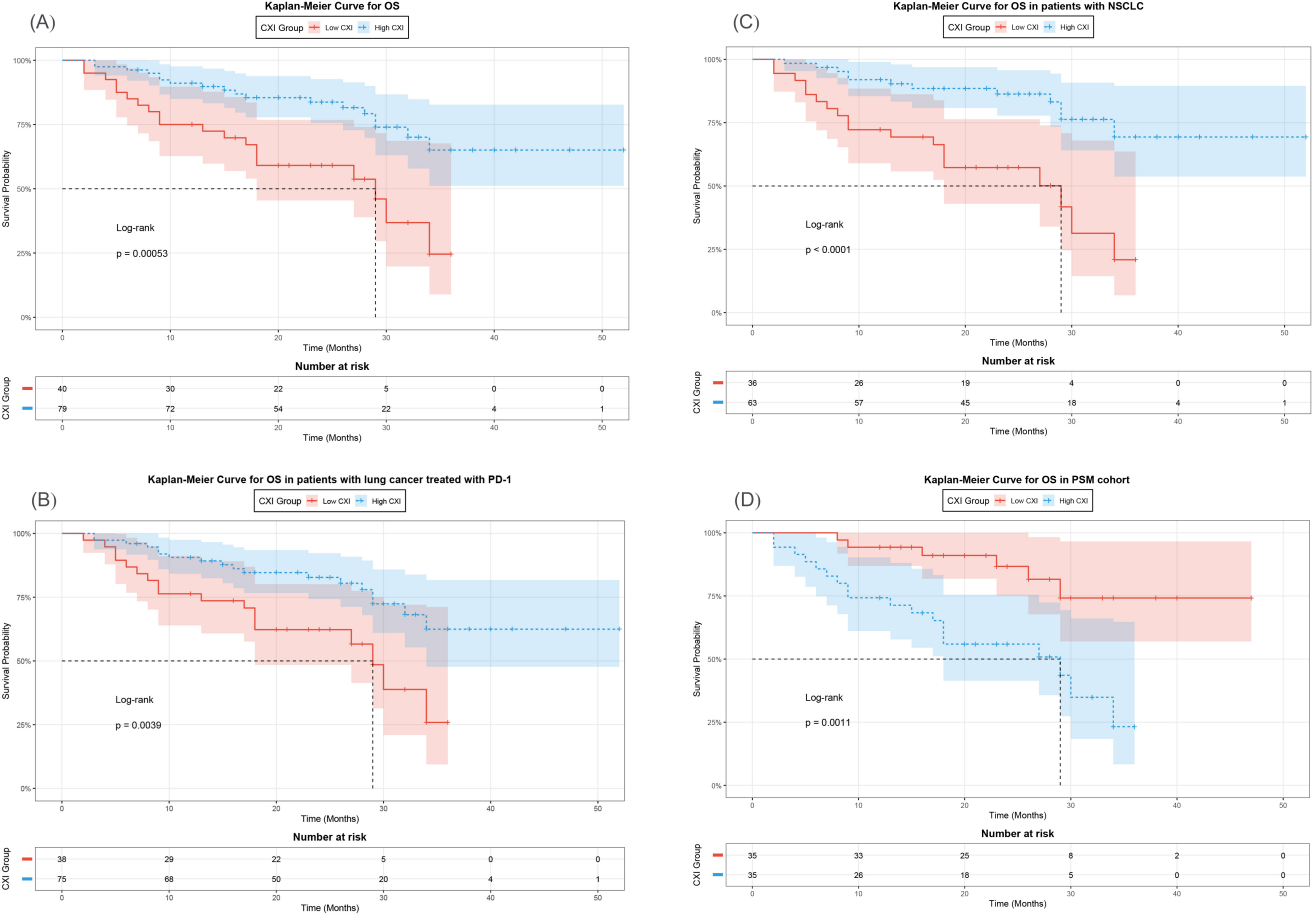
Kaplan-Meier curves of overall survival (OS) according to the cachexia index (CXI) in lung cancer patients treated with immunotherapy: total cohort **(A)**, patients receiving programmed cell death protein 1 (PD-1) inhibitors **(B)**, patients with non-small cell lung cancer (NSCLC) **(C)**, and the PSM cohort **(D)**.

The Kaplan-Meier survival curves of OS according to CXI are shown in Figure 1A. The patients with low CXI had a shorter survival time than the patients with high CXI (*P* < 0.001). The 3-year survival rates were 24.6% (95% CI: 8.9%–67.7%) and 65.1% (95% CI: 51.2%–82.7%) in the low and high groups, respectively. In the patients with NSCLC, the 3-year survival rates were 20.9% (95% CI: 6.9%–63.6%) and 69.3% (95% CI: 53.7%–89.5%) in the low and high groups, respectively (Figure 1C). Similar results were obtained in patients treated with PD-1, with 3-year survival rates of 25.8% (95% CI: 9.4%–71.1%) and 62.4% (95% CI: 47.7%–81.7%) in patients with low CXI and those with high CXI (Figure 1B). In the PSM cohort, the estimated 3-year OS rate was significantly lower in the low CXI group than in the high CXI group (23.2% vs. 74.2%, *P* = 0.001) (Figure 1D).

### 3.3 Univariate and multivariable analyses

The results of the Cox analysis are presented in Table 2 and Table 3. Univariate analysis showed that ALB, NLR, and CXI were significant predictors of overall survival time in patients treated with ICIs. In the multivariate analyses, CXI was associated with worse OS (HR = 3.11; 95% CI, 1.52–6.37; *P* < 0.001). In the PSM cohort, similar results were obtained. In the multivariate analyses, low CXI predicted high mortality (HR = 5.55; 95% CI, 1.86–16.61; *P* < 0.001). These results indicated that CXI was an independent risk factor for OS. We performed additional stratified analyses restricted to patients with NSCLC, stage IV disease, and those receiving PD-1 inhibitors. In patients with NSCLC, low CXI remained an independent poor prognostic factor for shorter OS compared with high CXI (HR = 4.37, 95% CI 1.84–10.42, *P* = 0.001). The prognostic significance of CXI was consistently observed in the stage IV subgroup (HR = 3.17, 95% CI 1.36–7.38, *P* = 0.007) and PD-1 subgroup (HR = 2.45, 95% CI: 1.17–5.13, *P* = 0.017) (Table 3).

**TABLE 2:** Univariate and multivariate Cox analyses.

Characteristic	Level	Total cohort				PSM cohort			
		Univariate analysis HR (95% CI)	*P*-value	Multivariable analysis HR (95% CI)	*P*-value	Univariate analysis HR (95% CI)	*P*-value	Multivariable analysis HR (95% CI)	*P*-value
Age	Continuous	1.03 (1.00–1.06)	0.098	1.02 (0.99–1.06)	0.141^#^	1.01 (0.97–1.05)	0.646	1.01 (0.97–1.06)	0.631^#^
Gender	Male	Reference		Reference		Reference		Reference	
	Female	0.92 (0.47–1.79)	0.798	0.95 (0.28–3.24)	0.934^#^	0.76 (0.33–1.71)	0.503	1.23 (0.25–6.12)	0.803^#^
BMI	23.9	Reference		Reference		Reference		Reference	
	<23.9	1.67 (0.88–3.17)	0.115	2.25 (1.10–4.63)	**0.027** ^#^	1.23 (0.56–2.72)	0.604	2.09 (0.80–5.48)	0.133^#^
Nutritional risk	Negative	Reference		Reference		Reference		Reference	
	Positive	1.55 (0.80–3.01)	0.198	1.56 (0.75–3.25)	0.237^#^	1.63 (0.74–3.61)	0.229	1.97 (0.77–5.02)	0.157^#^
Smoking	No	Reference		Reference		Reference		Reference	
	Yes	1.35 (0.70–2.60)	0.375	1.01 (0.31–3.37)	0.982^#^	1.11 (0.50–2.44)	0.803	0.64 (0.13–3.13)	0.580^#^
Stage	II-III	Reference		Reference		Reference		Reference	
	IV	2.05 (0.86–4.93)	0.107	2.86 (1.15–7.10)	**0.024** ^#^	1.49 (0.56–3.99)	0.428	2.41 (0.81–7.16)	0.115^#^
Surgery	No	Reference		Reference		Reference		Reference	
	Yes	0.48 (0.12–2.02)	0.318	0.59 (0.14–2.62)	0.491^#^	0.33 (0.04–2.43)	0.275	0.29 (0.04–2.42)	0.255^#^
Type of pathology	NSCLC	Reference		Reference		Reference		Reference	
	SCLC	1.01 (0.45–2.30)	0.973	1.03 (0.38–2.74)	0.959^#^	0.71 (0.21–2.39)	0.579	0.45 (0.09–2.28)	0.333^#^
Type of ICIs	PD-1	Reference		Reference		Reference		Reference	
	PD-L1	0.95 (0.23–3.97)	0.945	1.24 (0.24–6.32)	0.795^#^	1.20 (0.28–5.10)	0.804	2.34 (0.36–15.29)	0.375^#^
ALB	Continuous	0.91 (0.86–0.97)	**0.001**	/		0.93 (0.87–0.99)	**0.023**	/	
NLR	Continuous	1.12 (1.05–1.20)	**0.001**	/		1.09 (1.00–1.19)	**0.038**	/	
SMI	Continuous	0.97 (0.94–1.01)	0.180	/		0.98 (0.94–1.03)	0.524	/	
CXI	Continuous	0.998 (0.997–0.999)	**0.002**	0.998 (0.997–1.000)	**0.007** ^##^	0.998 (0.997–1.000)	**0.027**	0.998 (0.997–1.000)	**0.002** ^##^
	High	Reference		Reference		Reference		Reference	
	Low	2.98 (1.56–5.66)	**<0.001**	3.11 (1.52–6.37)	**<0.001^#^**	4.11 (1.64–10.31)	**<0.001**	5.55 (1.86–16.61)	**<0.001^#^**

^#^Adjusted for age, gender, BMI, nutritional risk, smoking, stage, surgery. Type of pathology, type of ICIs and CXI (low vs. high). ^##^Adjusted for age, gender, BMI, nutritional risk, smoking, stage, surgery. Type of pathology, type of ICIs and CXI (continuous). HR, hazard ratio; CI, confidence interval; BMI, body mass index; ALB, albumin; NLR, neutrophil to lymphocyte ratio; SMI, skeletal muscle index CXI, cachexia index; SCLC, small cell lung cancer; NSCLC, non-small-cell lung cancer; ICIs, immune checkpoint inhibitors; PD-L1, programmed cell death ligand 1; PD-1, programmed cell death protein 1. The statistically significant values were formatted in bold.

**TABLE 3:** Univariate and multivariate Cox analyses in patients with NSCLC, stage IV and PD-1 inhibitors.

Characteristic	Level	Patients with NSCLC				Patients with stage IV				Patients with PD-1 inhibitors			
		Univariate analysis HR (95% CI)	*P*-value	Multivariable analysis HR (95% CI)	*P*-value	Univariate analysis HR (95% CI)	*P*-value	Multivariable analysis HR (95% CI)	*P*-value	Univariate analysis HR (95% CI)	*P*-value	Multivariable analysis HR (95% CI)	*P*-value
Age	continuous	1.01 (0.98–1.05)	0.368	1.00 (0.96–1.03)	0.812^#^	1.03 (1.00–1.06)	0.088	1.02 (0.99–1.06)	0.160^#^	1.03 (1.00–1.06)	0.087	1.02 (0.99–1.06)	0.132^#^
Gender	Male	Reference		Reference		Reference		Reference		Reference		Reference	
	Female	0.87 (0.41–1.86)	0.724	0.45 (0.10–2.18)	0.325^#^	0.93 (0.46–1.91)	0.848	0.82 (0.21–3.19)	0.769^#^	0.93 (0.46–1.85)	0.828	0.96 (0.28–3.36)	0.954^#^
BMI	≥ 23.9	Reference		Reference		Reference		Reference		Reference		Reference	
	<23.9	1.53 (0.73–3.21)	0.115	2.76 (1.17–6.51)	**0.021** ^#^	1.33 (0.66–2.68)	0.418	1.85 (0.83–4.13)	0.133^#^	1.25 (0.62–2.51)	0.528	1.67 (0.76–3.69)	0.206^#^
Nutritional risk	Negative	Reference		Reference		Reference		Reference		Reference		Reference	
	Positive	1.75 (0.85–3.62)	0.130	1.67 (0.75–3.70)	0.207^#^	1.58 (0.76–3.31)	0.224	1.42 (0.61–3.30)	0.415^#^	1.73 (0.87–3.45)	0.118	1.56 (0.75–3.26)	0.236^#^
Smoking	No	Reference		Reference		Reference		Reference		Reference		Reference	
	Yes	1.57 (0.75–3.27)	0.234	2.34 (0.50–10.99)	0.280^#^	1.33 (0.65–2.70)	0.431	1.00 (0.25–3.96)	1.000^#^	1.30 (0.66–2.56)	0.442	1.02 (0.31–3.38)	0.976
Stage	II-III	Reference		Reference		Reference		Reference		Reference		Reference	
	IV	1.60 (0.66–3.92)	0.300	1.98 (0.74–5.29)	**0.173** ^#^	-	-	-	-	1.67 (0.69–4.01)	0.324	2.29 (0.90–5.78)	0.081**^#^**
Surgery	No	Reference		Reference		Reference		Reference		Reference		Reference	
	Yes	0.53 (0.12–2.22)	0.381	0.76 (0.17–3.41)	0.723^#^	0.55 (0.13–2.32)	0.415	0.64 (0.14–2.85)	0.557^#^	0.49 (0.12–2.04)	0.324	0.57 (0.13–2.50)	0.454^#^
Type of pathology	NSCLC	Reference		Reference		Reference		Reference		Reference		Reference	
	SCLC	/		/	/	1.23 (0.53–2.83)	0.635	1.42 (0.49–4.13	0.518^#^	1.11 (0.46–2.68)	0.811	1.25 (0.49–3.23)	0.642^#^
Type of ICIs	PD-1	/		/		Reference		Reference		/		/	
	PD-L1	1.58 (0.21–11.79)	0.658	8.56 (0.63–117.17)	0.108^#^	3.84 (0.90–16.37)	0.069	1.66 (0.27–10.14)	0.583^#^	/		/	
ALB	Continuous	0.90 (0.84–0.95)	**<0.001**	/		0.93 (0.88–0.99)	**0.023**	/		0.91 (0.86–0.97)	**0.002**	/	
NLR	Continuous	1.15 (1.07–1.24)	**<0.001**	/		1.12 (1.05–1.21)	**0.001**	/		1.11 (1.04–1.20)	**0.003**	/	
SMI	Continuous	0.97 (0.93–1.02)	0.180	/		0.97 (0.93–1.01)	0.103	/		0.98 (0.94–1.02)	0.310	/	
CXI	Continuous	0.997 (0.996–0.999)	**0.001**	0.997 (0.995–0.999)	**0.003** ^##^	0.999 (0.998–1.000)	**0.010**	0.999 (0.997–1.000)	**0.019** ^##^	0.999 (0.998–1.000)	**0.007**	0.999 (0.998–1.000)	**0.020^##^**
	High	Reference		Reference		Reference		Reference		Reference		Reference	
	Low	3.93 (1.89–8.16)	**<0.001**	4.37 (1.84–10.42)	**0.001^#^**	2.79 (1.38–5.63)	**0.004**	3.17 (1.36–7.38)	**0.007^#^**	2.56 (1.33–4.96)	**0.005**	2.45 (1.17–5.13)	**0.017^#^**

^#^Adjusted for age, gender, BMI, nutritional risk, smoking, stage, surgery. Type of pathology, type of ICIs and CXI (low vs. high). ^##^Adjusted for age, gender, BMI, nutritional risk, smoking, stage, surgery. Type of pathology, type of ICIs and CXI (continuous). HR, hazard ratio; CI, confidence interval; BMI, body mass index; ALB, albumin; NLR, neutrophil to lymphocyte ratio; SMI, skeletal muscle index CXI, cachexia index; SCLC, small cell lung cancer; NSCLC, non-small-cell lung cancer; ICIs, immune checkpoint inhibitors; PD-L1, programmed cell death ligand 1; PD-1, programmed cell death protein 1. The statistically significant values were formatted in bold.

### 3.4 Development of a nomogram model for OS

**FIGURE 2:**
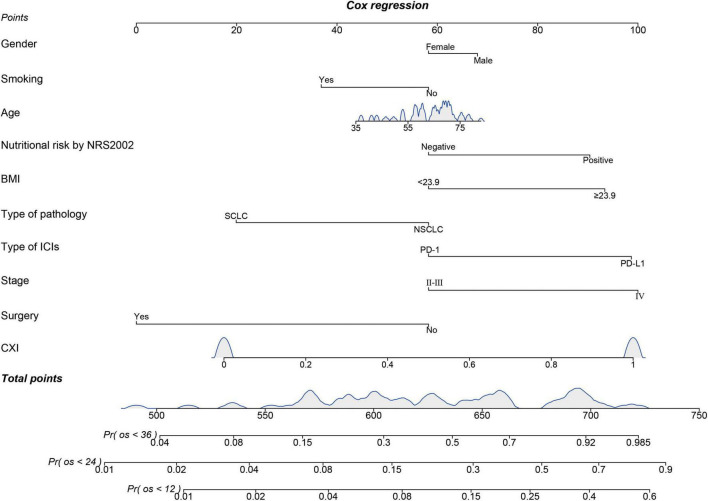
Prognostic nomogram for overall survival (OS) time prediction based on cachexia index (CXI) in lung cancer patients treated with immunotherapy.

A nomogram model was developed based on CXI to predict OS in patients treated with ICIs. All factors in the multivariate Cox regression analysis were used to establish the nomogram model, including age, BMI, CXI, gender, nutritional risk, smoking, stage, type of pathology, and type of ICIs. The nomogram synthesizes a cumulative score by summing the weighted point values assigned to these prognostic factors. For each variable, healthcare providers first identify the patient’s corresponding value on its respective axis, project it vertically onto the predefined points scale, and then then sum the points to obtain a total score. This total score is subsequently projected downward along the vertical axis to estimate the likelihood of overall survival at 1-, 2-, and 3-year follow-up (Figure 2).

### 3.5 The relationship between SMI and CXI

**FIGURE 3:**
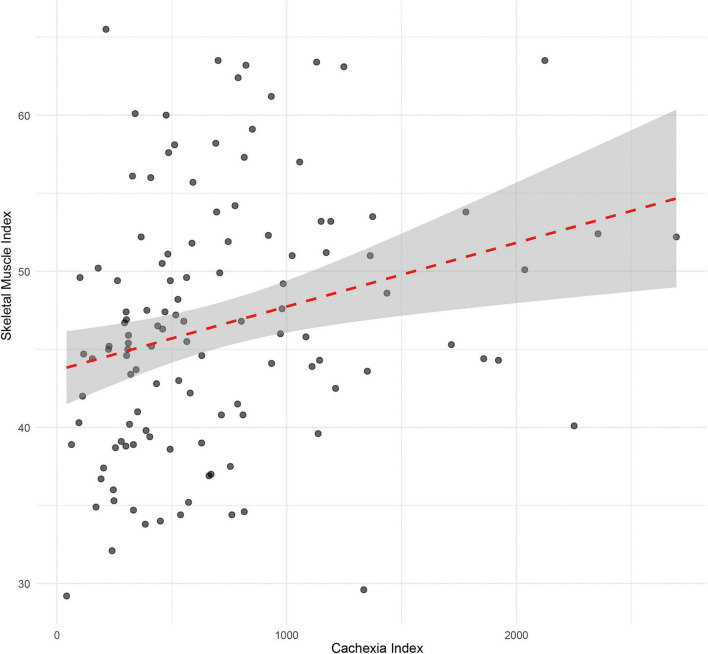
Scatter plot showing the correlation between cachexia index (CXI) and skeletal muscle index (SMI).

To examine the relationship between CXI and SMI, Spearman’s rank correlation analysis was performed. The results indicated a statistically significant weak to moderate positive correlation between the two variables (ρ = 0.328, *p* = 0.0003). This suggests that as CXI increases, SMI tends to increase correspondingly, although the monotonic association is not particularly strong (Figure 3).

## 4 Discussion

This study investigated the prognostic value of CXI in patients with lung cancer treated with immune checkpoint inhibitors. The results showed that patients with low CXI had shorter overall survival than those with high CXI. Multivariable Cox analysis also demonstrated that CXI was an independent prognostic factor for OS in these patients. Additionally, this study found that CXI was correlated with SMI, showing a statistically significant weak-to-moderate positive correlation.

The results of the present study were consistent with the findings from numerous previously published studies. A retrospective study of 81 patients with cervical cancer treated with radiotherapy indicated that pretreatment CXI was an independent prognostic factor (HR = 2.26) (11). A retrospective study of 267 patients with DLBCL who received R-CHOP chemotherapy was conducted by Go et al., and their findings indicated that CXI functioned as a predictive biomarker for survival, treatment response, treatment-related adverse events, and treatment adherence in this population (12). Another study indicated that CXI has good prognostic value in patients with gastric cancer. Furthermore, its prognostic value remained significant in subgroup analyses of different sexes, ages, cachexia statuses, BMIs, and TNM stages (13). These findings were further supported by in a single-center prospective study, which showed that OS in the patients with low CXI was significantly shorter than that in the patients with high CXI (14).

Similar results were also obtained in lung cancer. In a retrospective study of 145 patients, multivariate analysis demonstrated that pretreatment CXI was an independent predictor of OS (HR = 2.34) and PFS (HR = 2.38) in patients with lung metastases after stereotactic radiotherapy (15). Data from other studies have showed that patients with low CXI had significantly worse PFS and OS in both metastatic NSCLC and SCLC (16, 17). The present study is the first to explore the prognostic value of CXI in patients with lung cancer treated with ICIs. As ICIs are widely recommended in clinical practice for the treatment of lung cancer, these results indicate that CXI can also be used to identify patients requiring additional therapies during immunotherapy (14).

The reason why CXI acts as a predictor in this population can be explained as follows based on the three parameters used to calculate the index. Firstly, previous studies have shown that skeletal muscle at the L3 level on CT is associated with whole-body muscle mass. SMI at the L3 level was also recommended for use in diagnosing sarcopenia. Low muscle mass and sarcopenia have been proven to be associated with worse clinical outcomes (18). Secondly, ALB is associated with the nutritional status of patients with cancer. One published study showed that the pretreatment levels of ALB were significantly associated with survival in patients with advanced oesophageal cancer who were treated with ICIs (19). Malnutrition is also a strong negative predictor of overall survival time in cancer (20). Moreover, results from a prospective cohort study indicated that CXI demonstrated 100% sensitivity for diagnosing malnutrition (21). Furthermore, as the NLR is an inflammatory parameter, numerous studies have shown that it acts as a prognostic factor for OS in cancer. The NLR serves as a systemic indicator of the balance between the pro-tumor inflammatory response and the anti-tumor immune response within the tumor microenvironment. As a hallmark of cancer, inflammation was associated with cancer initiation, progression and metastasis (22). The results from an umbrella review also showed that a high NLR was associated with poor cancer prognosis (23). Similarly, a retrospective study also showed that low NLR characteristics predicted better OS in patients with advanced Epidermal Growth Factor Receptor -mutant non-small cell lung cancer receiving anti-PD-1 inhibitors (24).

The results also indicated that a statistically significant weak to moderate positive correlation was found between CXI and SMI (Spearman’s ρ = 0.328, *P* < 0.001). As SMI is a component of the CXI formula (CXI = SMI × ALB/NLR), a baseline correlation is mathematically expected. However, clinically, SMI primarily serves as a representative measure of whole-body muscle mass, whereas CXI reflects the complex, multifactorial syndrome of cachexia. Sarcopenia is primarily driven by aging, diagnosed via criteria integrating muscle strength, mass, and/or physical performance (25), while cachexia is a disease-related metabolic disorder characterized by involuntary weight loss, non-selective atrophy of both type I and II muscle fibers and severe systemic inflammation (5). Both syndromes predicting adverse outcomes in older patients with cancer but differing in intervention responsiveness (26). Sarcopenia benefits from resistance training and high-protein nutrition, while cachexia requires multimodal strategies with limited FDA-approved pharmacological options (27). This weak correlation suggests cachexia cannot be fully explained by low muscle mass alone. Rather, the inclusion of albumin and the neutrophil-to-lymphocyte ratio allows CXI to provide complementary prognostic information, capturing the systemic inflammatory and nutritional derangements that extend beyond the loss of muscle mass.

This study has several limitations. First, it was a retrospective, single-center study with the risk of selection bias, which may compromise the generalizability of the results. Second, nutritional and other interventions implemented during the treatment could have exerted a confounding effect on clinical outcomes; however, these data were not collected in the present study. Third, the use of telephone-based follow-up for a subset of patients may have led to inaccuracies in verifying mortality status. Fourth, the relatively small sample size potentially undermines the statistical robustness and reliability of the study’s findings. Fifth, the lack of assessments for PD-L1 expression and TMB further impairs the validity of the prognostic conclusions drawn from this analysis. Sixth, this investigation did not evaluate other clinical outcomes, such as progression-free survival and the incidence of treatment-related toxicities. Finally, this study could not compare low CXI with clinically diagnosed cachexia to investigate the diagnostic value of CXI for cancer cachexia. Published study have shown that CXI acted as a potential and useful tool to diagnose cancer cachexia. A cross-sectional study had shown that CXI was independently associated with Asian Working Group for Cachexia-defined cachexia (OR=̃ 0.98, 95% CI: 0.97–0.99, *p* < 0.001) (28). Therefore, more prospective studies are needed in the future to verify the diagnostic value of CXI for cachexia.

## 5 Conclusion

In patients with lung cancer treated with ICIs, CXI serves as a strong prognostic factor for OS. Concurrently, SMI is only weakly to moderately correlated with CXI in this population. Notably, CXI holds promise as a potential tool for diagnosing cancer cachexia in patients with lung cancer receiving immunotherapy. In clinical practice, the index can be utilized to predict OS and assist in clinical decision-making. Further large-sample prospective trials are warranted to validate the diagnostic value of CXI for cancer cachexia and to determine the optimum cutoff values for different cancer types and stages of cancer cachexia.

## Data Availability

The datasets presented in this article are not readily available because due to the nature of this research, participants of this study did not agree for their data to be shared publicly, so supporting data is not available. Requests to access the datasets should be directed to XJ, jinxinrd@alumni.hust.edu.cn.

## Supplementary material

Supplementary Figure 1 Time- dependent receiver operating characteristic (ROC) analysis for the cachexia index (CXI).
