# Supplementary figures and images for: The prognostic value of cachexia index in patients with lung cancer treated with immune checkpoint inhibitors: a propensity score matching analysis
Source: Front Nutr. 2026 Jul 20;13:1810531. doi: 10.3389/fnut.2026.1810531 (PMC13430999; doi:10.3389/fnut.2026.1810531)

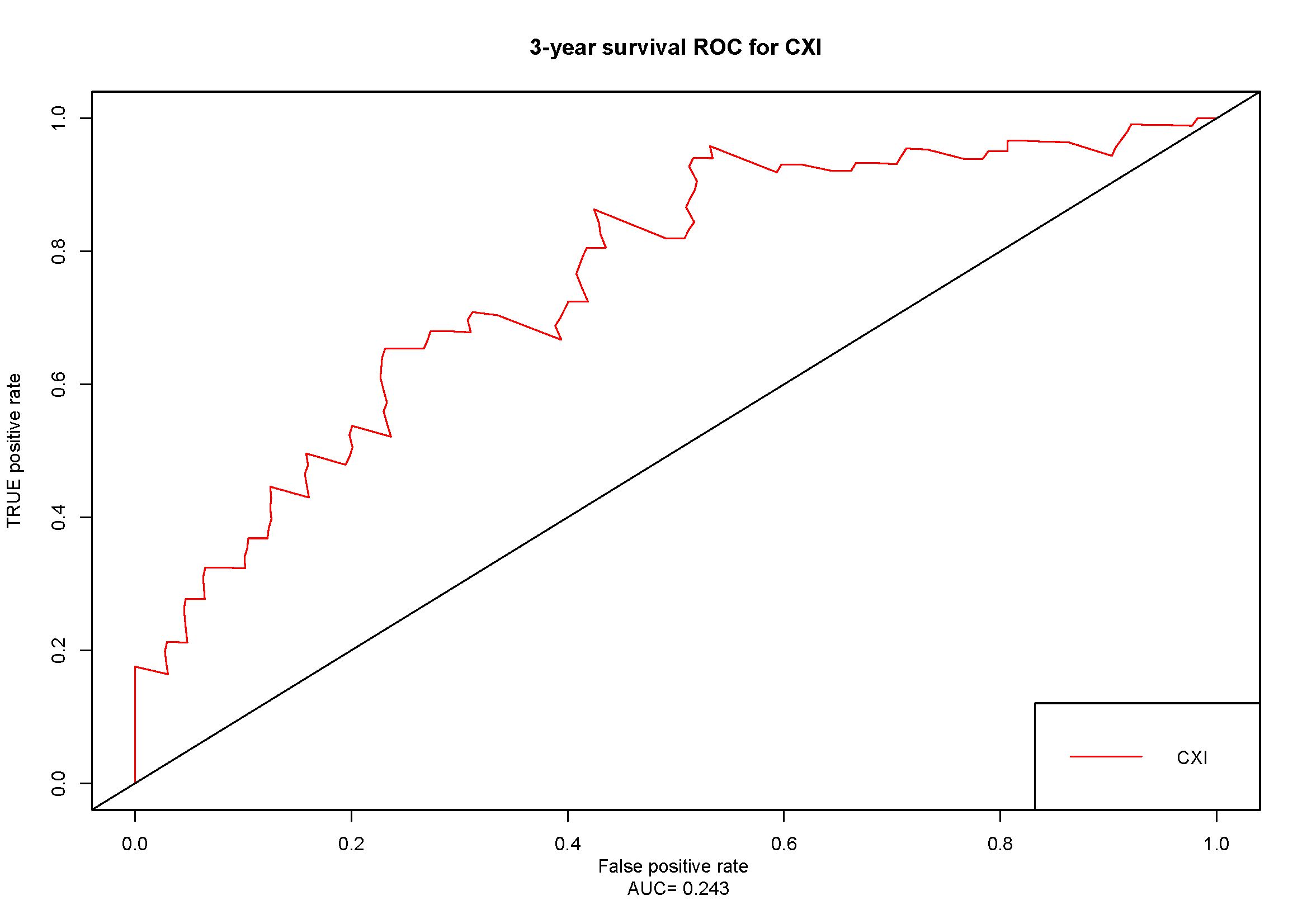

Supplement: Supplementary Figure 1 — Time- dependent receiver operating characteristic (ROC) analysis for the cachexia index (CXI). [file Image_1.tiff]
